# Effects of Deep Brain Stimulation on Adductor Laryngeal Dystonia

**DOI:** 10.1002/lary.70441

**Published:** 2026-02-16

**Authors:** Rita R. Patel, Maggie Stall, Stacey Halum, Benjamin Anthony, Noah Parker, Morgan Reese, David Kareken, Stephanie Dickinson, Thomas C. Witt, David A. Purger, Kunal Gupta, S. Elizabeth Zauber

**Affiliations:** ^1^ Department of Otolaryngology Head and Neck Surgery Indiana University School of Medicine Indianapolis Indiana USA; ^2^ Department of Speech, Language and Hearing Sciences Indiana University Bloomington Indiana USA; ^3^ Department of Medicine Indiana University School of Medicine Bloomington Indiana USA; ^4^ Department of Neurology Indiana University School of Medicine Indianapolis Indiana USA; ^5^ Department of Epidemiology and Biostatistics Indiana University School of Public Health Bloomington Indiana USA; ^6^ Department of Neurological Surgery Indiana University School of Medicine Indianapolis Indiana USA; ^7^ Department of Neurosurgery Medical College of Wisconsin Milwaukee Wisconsin USA

**Keywords:** adductor laryngeal dystonia, adductor spasmodic dysphonia, deep brain stimulation, GPi, VIM

## Abstract

**Objective:**

To examine the effects of globus pallidus interna (GPi) and ventral intermediate nucleus of the thalamus (VIM) deep brain stimulation (DBS) on patients with adductor laryngeal dystonia (ADLD).

**Methods:**

Seven patients with ADLD underwent DBS (GPi = 4; VIM = 3) surgery. Postoperative voice testing was performed after stable DBS programming. Primary outcome measures included tremor rate, extent of fundamental frequency/intensity modulation, percentage of voicing, duration/number of voice breaks, and cepstral peak prominence. Linear mixed effects models tested voice improvement after GPi and VIM surgery, with significance determined after controlling for multiple comparisons.

**Results:**

GPi‐DBS showed trends for improved percentage of voicing, duration of voice breaks, number of voice breaks, and extent of intensity modulation pre–post within‐group with large effect size. VIM‐DBS showed trends for improved tremor rate within‐group with large effect size. Between‐group comparison showed greater improvement in percentage voicing and extent of intensity modulation in patients with GPi‐DBS compared to VIM‐DBS, whereas tremor rate showed greater improvement after VIM‐DBS compared to GPi‐DBS. Duration of voice breaks showed more improvement in GPi than VIM but it did not achieve statistical significance after multiple comparison adjustments.

**Conclusions:**

Objective acoustic voice analyses provide preliminary, target‐specific patterns that warrant confirmation of bilateral GPi‐DBS for patients with ADLD and bilateral VIM‐DBS for those with both ADLD and vocal tremor. Future research with larger sample sizes, along with investigations into the neuronal mechanisms underlying laryngeal neuromodulation, is needed to further evaluate the role of DBS in treating ADLD.

**Level of Evidence:**

3.

## Graphical abstract

This study evaluated how globus pallidus interna (GPi) versus ventral intermediate nucleus (VIM) deep brain stimulation (DBS) affects objective voice measures in patients with adductor laryngeal dystonia. GPi‐DBS was associated with greater improvements in voicing, voice breaks, and intensity modulation, while VIM‐DBS showed greater improvement in tremor rate.

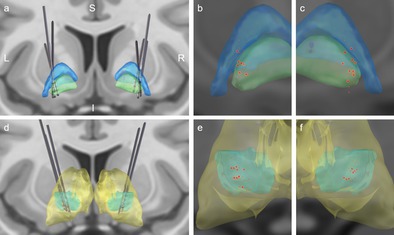

## 1 Introduction

Laryngeal dystonia (LD), also known as spasmodic dysphonia [1], is a movement disorder involving uncontrollable, repetitive muscle contractions of the larynx. LD is a form of isolated focal dystonia of the laryngeal adductor and/or abductor muscles. Task specificity is a hallmark of the disorder, with speaking ability being greatly affected while other vocal activities such as whisper remain intact [1]. Adductor laryngeal dystonia (ADLD) is the most common form of LD, accounting for 80%–90% of cases. The standard treatment of LD involves botulinum toxin injections into the laryngeal muscles, which provide temporary relief of vocal symptoms and require repeated injections to maintain therapeutic benefit [2, 3]. Among patients who respond to botulinum toxin, only around 30% experience significant improvements [4, 5], while approximately 51% report adverse effects, such as breathiness [5].

Dystonia, such as LD, is largely described as a neurological disorder of the basal ganglia [6, 7], however recent neuroimaging studies suggest that LD is a functional and structural neural network disorder [8]. It is characterized by abnormal neurotransmission and a loss of normal inhibitory tone [9]. Deficient GABAergic neurotransmission has been observed as the specific mechanism for the loss of inhibition seen with LD [10]. This occurs as a consequence of abnormal dopaminergic signaling, generating a hyperexcitable basal ganglia‐thalamo‐cortical circuit characterized by hyperfunctional direct (excitatory) and hypofunctional indirect (inhibitory) pathways in the basal ganglia, resulting in dystonic muscle movements [7, 11, 12]. Recent studies have demonstrated altered structural and functional connectivity across a broader network of brain regions beyond the basal ganglia, including hyperexcitability of the speech motor control network and somatosensory feedback networks, and reductions in sensorimotor network connectivity in LD [1, 10, 13]. These functional alterations are linked with structural changes in areas of the cerebello‐thalamo‐cortical network, known to relay motor cortical activity, including the inferior parietal and sensorimotor cortices, putamen, and cerebellum [13, 14]. Targeting these specific alterations in network dynamics may be essential for effectively addressing the underlying mechanisms of dystonia.

Deep brain stimulation (DBS) of the ventral intermediate nucleus of the thalamus (VIM) is a well‐established treatment for essential tremor and tremor‐dominant Parkinson disease [15], while DBS of the globus pallidus pars interna (GPi) is a recognized treatment approach for treating focal dystonia [16, 17]. In our recent paper involving 10 patients with essential tremor affecting both the hands and the voice, we demonstrate that bilateral VIM‐DBS effectively and significantly improved voice quality by reducing the rate of vocal tremor [18]. Additionally, in a patient with ADLD, we used blinded quantitative voice analysis to show that bilateral GPi‐DBS was effective in reducing voice breaks and improving both voice and overall speech intelligibility [19]. Here, we build upon our previous work by reporting our recent experience with bilateral GPi‐DBS and VIM‐DBS on patients with ADLD. We hypothesized that, relative to pre‐operative performance, GPi‐DBS would show trends toward greater improvement in ADLD‐specific outcomes (percentage voicing, number and duration of voice breaks, CPP), whereas VIM‐DBS would show trends toward greater improvement in tremor‐specific outcomes (tremor rate, extent of frequency modulation and intensity modulation) in patients with ADLD and comorbid vocal tremor.

## 2 Materials and Methods

### 2.1 Study Patients

A cohort of four patients with ADLD was recruited for bilateral GPi‐DBS; each patient consented to treatment and signed an IRB‐approved informed consent form. Inclusion criteria required that the patients had a diagnosis of ADLD with or without vocal tremor, confirmed through routine clinical examination by a laryngologist, speech language pathologist, and neurologist. Only native speakers of American English were included due to well‐documented differences in voice and neural signals between native and non‐native speakers. To ensure no residual effects on voice quality of botulinum toxin injection, patients had to be at least 3 months postinjection and fully symptomatic. Exclusion criteria included history of stroke, brain surgery, dementia, or other neurological disorders besides tremor and focal dystonia; prior laryngeal framework surgery, and presence of ferromagnetic and/or cardiac implants, and patients who were asymptomatic due to previous treatment with botulinum toxin injections into the laryngeal muscles. Preoperative voice testing and neuropsychological testing were performed 3–6 months prior to DBS surgery. Postoperative assessments using the same tests were conducted 6–9 months after surgery, once stable DBS settings were achieved by the neurologist.

A second cohort of patients had coexisting LD and essential tremor and underwent bilateral VIM‐DBS (*n* = 3) for medically refractory, debilitating hand tremor (Table 1). These patients are from our prior study and were used as a comparison group [18]. They consented to pre‐ and postoperative voice testing to study the impact of VIM‐DBS on LD. Since VIM‐DBS for hand tremor is standard of care, they did not undergo postoperative neuropsychological testing (except for limited examination of psychomotor speed and verbal fluency preoperatively).

**TABLE 1:** Demographics of patient cohort.

Patient #	Sex	Age at surgery	Handedness	Primary diagnosis	Symptom duration	Additional diagnosis	Head tremor	Extremity tremor	DBS surgery
1	F	73	R	ADLD/vocal tremor	22 years		No	Minimal	GPi
2	M	58	L	ADLD	27 years	Blepharospasm Mild OMD	No	No	GPi
3	F	76	R	ADLD/vocal tremor	9 years	Cervical Dystonia	No	L > R Mild	GPi
4	F	70	R	ADLD/vocal tremor	5 years	DT	No	EB Moderate	GPi
5	F	69	L	ADLD/ET/vocal tremor	28 years		Yes Mild	EB Moderate	VIM
6	F	70	L	ADLD/ET/vocal tremor	12 years		Yes Moderate	R > L Moderate	VIM
7	F	82	R	ADLD/ET/vocal tremor	23 years		Yes Moderate	Severe L > R	VIM

*Note*: Sex: Female (F), male (M). Handedness: Left (L), right (R). Diagnoses: Essential tremor (ET), dystonic tremor (DT), laryngeal dystonia (LD), oromandibular dystonia (OMD). Extremity tremor: Right worse than left (R > L), left worse than right (L > R), equal bilaterally (EB). DBS, deep brain stimulation surgery; GPi, globus pallidus pars interna; VIM, ventral intermediate nucleus of the thalamus. Patient #2 has mild OMD which did not affect articulation/voice and was only bothersome for cosmetic effects.

A total of 6/7 patients had vocal tremor. 5/7 patients presented with isolated dystonia and 1/7 with additional craniocervical dystonia.

### 2.2 Acoustic Voice Recording Setup

Voice recordings were collected in a quiet clinic room with acoustic foam paneling at the IU Health Voice Center. Acoustic recordings were captured at 44.1 kHz on the Computerized Speech Lab (Model 4500, PENTAXMedical, Montvale New Jersey) with a Shure Beta 53 headset microphone at a fixed mouth‐to‐microphone distance [20] of 1 in. at an angle of 45°. We recorded three trials each of the following utterances: sustained vowel /a:/ for 4 s, and an all voiced‐sentence, “Early one morning a man and a woman were ambling along a one‐mile lane running near rainy island avenue” [21].

The following parameters were computed from the middle 1 s of the sustained phonation: cepstral peak prominence (CPP), percentage voicing, tremor rate (hertz), extent of fundamental frequency modulation (%), and extent of intensity modulation (%) for three trials. The CPP (decibels) is a dominant “rahmonic” within the cepstrum [22], which measures the relative contributions of periodic and aperiodic components of the voice signal. Percentage voicing is defined as the proportion of time spent in voicing within 1 s. The tremor rate was determined by counting the total number of cycles of frequency modulations in 1 s [23, 24]. The extent of frequency/intensity modulation in percentage was calculated by dividing the range of the frequency/intensity modulation with the sum of the maximum and minimum frequency/intensity and then multiplied by 100 [23, 24]. Duration (milliseconds) and number of voice breaks were counted from 4 s of sustained phonation [25, 26, 27]. Parameters of CPP, percentage voicing, duration/number of voice breaks are more distinguishing for LD, whereas tremor rate and extent of frequency/intensity modulation are more characteristic of vocal tremor.

### 2.3 Patient Self Perception of Voice Problem

All four patients who underwent GPi‐DBS completed the Voice Handicap Index‐30 scale [28].

### 2.4 Neuropsychological Testing

A preoperative neuropsychological battery (which includes tests of frontal‐executive function, language, intellectual ability, memory, and mood) was performed on all patients undergoing DBS surgery according to standard of care, and to rule out significant cognitive impairment/dementia. The preoperative test battery included the Mini Mental State Exam [29], Repeatable battery for the Assessment of Neuropsychological Status [30], Trial Making Tests [31], Controlled Oral word Fluency [32], and Modified Wisconsin Card Sorting Test [33]. A limited postoperative battery consisting of the Trail Making Tests [31] (parts A and B) and the Controlled Oral Word Association test [32] (i.e., letter fluency, both of which were included in the original clinical examination) was performed on patients 1–3 with GPi‐DBS since this target was new for this indication. In this preliminary study, only results from pre‐ and post‐tests where comparisons can be made are reported (in accordance with standard of care practices, a complete postoperative battery of neuropsychological testing is not conducted unless clinically indicated). Normative data for interpretation are from the Minnesota Older Adults Normative Study, with standard scores (mean = 10, SD = 3) converted to percentile ranges [34]. Here we refer to a clinically normal range as being above the 10th percentile, roughly corresponding to the “below average” appellation by Guilmette et al. [35].

### 2.5 DBS Surgery

DBS surgery was performed in two stages. Stage 1 involved awake, Leksell (Elekta) G‐frame‐based microelectrode recording (MER)–guided placement of bilateral DBS electrodes, with intraoperative radiographic confirmation of targeting accuracy. Stage 2 (placement of extension leads and infraclavicular internal pulse generator) was performed 2–3 weeks after stage 1. The choice of DBS system was determined by the neurology and neurosurgery teams and was chosen based on each patient's individual clinical need. Perioperative care and procedures were performed according to standard of care guidelines.

### 2.6 DBS Lead Reconstruction

DBS electrodes were reconstructed using the Lead‐DBS software package [36]. Preoperative imaging (T1‐weighted MRI, *n =* 6; fGATIR MRI, *n* = 1) was co‐registered to postoperative imaging (CT, *n =* 6; T1‐weighted MRI, *n* = 1) using a rigid, followed by affine, linear registration as implemented in the Advanced Normalization Tools package [37] (MRI to CT, *n =* 6) and the SPM12 package [38] (MRI to MRI, *n* = 1). Pre‐ and postoperative sequences were spatially normalized into MNI152NLin2009bAsym space [39] based on preoperative anatomic T1‐weighted or fGATIR MRI sequences using the SyN registration approach as implemented in Advanced Normalization Tools. Nonlinear deformation into template space was achieved in five stages: after two linear (rigid followed by affine) steps, a nonlinear (whole brain) SyN‐registration stage was followed by two nonlinear SyN‐registrations that consecutively focused on the area of interest as defined by subcortical masks in [40]. dB electrodes were automatically reconstructed in native and MNI space using the PaCER algorithm [41] (*n* = 6); in one patient, electrodes had to be localized manually using Lead‐DBS. Electrodes and active contacts were visualized in MNI space with overlaid anatomic structures derived from the DISTAL Minimal and Medium atlases [42, 43].

### 2.7 Statistical Analysis

Cohen's effect sizes were calculated for the within‐group changes (dz) pre‐ to post‐surgery as well as comparing the changes between GPi and VIM surgery types (Cohen's *d*) [44]. The Cohen's *d* (between group) and dz (within‐group) effect sizes provide an overall estimate of the standardized size of differences (mean/standard deviation), regardless of sample size or *p*‐values and are commonly interpreted as small = 0.2, medium = 0.5, and large = 0.8 [44].

Linear mixed effects models were used to determine whether patient's acoustic voice measurements improved after GPi‐ or VIM‐DBS surgery. Models for each outcome measure included factors of time (pre/post), group (GPi/VIM), the interaction between time and group, as well as a random effect to account for the correlation for each person over time (pre/post). Estimated mean differences (Est), standard errors (SE) and *p* values are reported for postoperative changes in voice quality within each group (pre–post) as well as differences between groups in their respective changes. The Benjamini–Hochberg FDR method was used to determine significance after controlling for multiple comparisons across the 15 within‐group comparisons and seven between‐group comparisons [45]. Additionally, Spearman correlations were performed to explore how improvements in Voice Handicap Index are correlated with changes in other outcome measures, using change scores calculated as post–pre, after first averaging the trials within time point for each person. Spearman correlations with *p*‐values from exact tests are used to reduce reliance on distributional assumptions in the context of small sample size. Alpha level was set at 0.05 (two‐sided). All analyses were performed using R Statistical Software (v4.2.2; R Core Team).

## 3 Results

**FIGURE 1:**
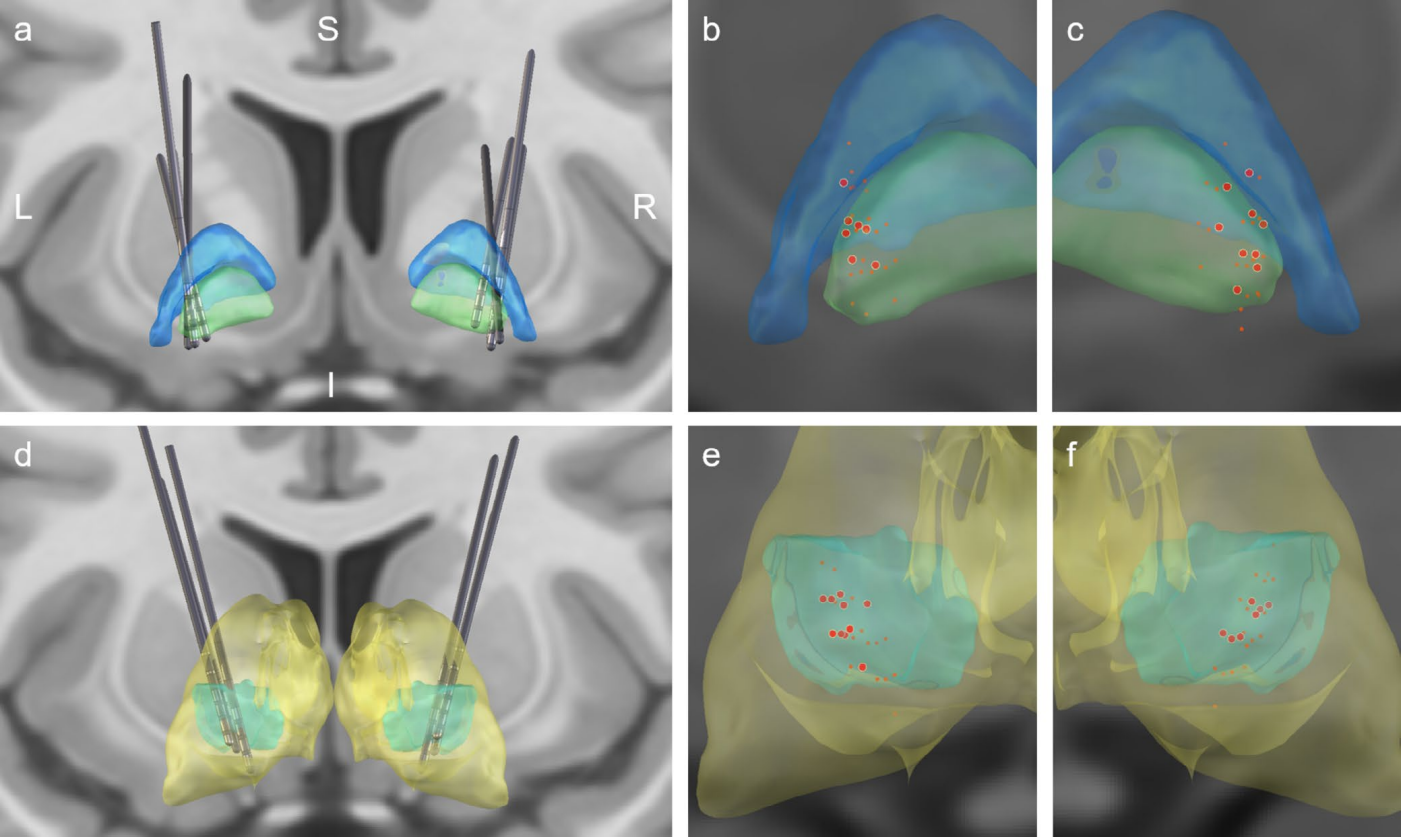
Location of deep brain stimulating (DBS) electrode placement normalized to a standard space. (a) DBS electrodes (silver; *n* = 4 per side) traversing in the bilateral globus pallidus pars externa (GPi; blue) and targeting globus pallidus pars interna (green). (b, c) Detailed view of electrode contacts (orange; *n* = 28 per side) in left (b) and right (c) GPi with active contacts highlighted (red with white outline). (d) DBS electrodes (silver; *n* = 3 per side) traversing thalamus (yellow) and targeting ventral intermediate nucleus of thalamus (VIM; teal). (e, f) Detailed view of electrode contacts (orange; *n* = 24 per side) in left (e) and right (f) VIM with active contacts highlighted (red with white outline). S = superior; I = inferior; L = left; *R* = right.

GPi was targeted with the following coordinates relative to the mid‐commissural point (MCP): 19.08 ± 0.5 mm lateral, 2.8 ± 0.6 mm anterior, and 2.6 ± 1.3 mm inferior (Table 2). Mean programming parameters for GPi‐DBS were amplitude 3.2 ± 1.1 mA, pulse width 105 ± 17.3 ms, and frequency 93.8 ± 39.0 Hz. VIM was targeted with the following coordinates relative to MCP: 12.8 ± 0.5 mm lateral, 6.0 ± 0.06 mm posterior, and 0.3 ± 0.6 mm inferior (Table 2, Figure 1a–f). Mean programming parameters for VIM‐DBS were amplitude 1.5 ± 0.0 mA, pulse width 60.0 ± 20.0 ms, and frequency 116.7 ± 15.3 Hz.

**TABLE 2:** Deep brain stimulation (DBS) settings at time of post‐operative vocal recording follow‐up.

Patient # and target	Lead type	Time post‐op (months)	Left					Right				
			Active contact (mA)	Active contact coordinates (LR, AP, SI)	Amplitude (mA)	Pulse width (μs)	Frequency (Hz)	Active contact (mA)	Active contact coordinates (LR, AP, SI)	Amplitude (mA)	Pulse width (μs)	Frequency (Hz)
1/GPi	MS	7	1b− (1.5) 2b− (1.5)	−22.6, −7.1, −2.8 −22.8, −5.6, −1.2	4.5	90	130	9c− (1.2) 10c− (1.2)	20.4, −6.4, −3.3 20.9, −5.0, −1.5	3.7	90	130
2/GPi	MS	6	1b− (1.0) 2b− (1.0)	−21.1, −7.5, −5.2 −21.6, −6.1, −3.7	3.5	120	60	9b− (1.1) 10b− (1.1)	21.3, −8.7, −6.0 21.6, −7.4, −4.4	3.0	120	60
3/GPi	MS	6	2b− (0.7)	−22.8, −7.9, −3.1	2.0	90	125	9c− (1.3) 10c− (1.3)	22.5, −7.4, −5.2 22.9, −6.5, −3.1	4.1	100	125
4/GPi	M	24	3—	−22.3, −4.5, −0.98	2.6	120	60	10− 11−	22.2, −6.4, −2.5 22.1, −4.8, −0.80	2.3	150	60
5/VIM	B	3	L2− ring L3− ring	−15.9, −17.4, 1.7 −16.5, −16.1, 3.5	1.5	40	130	L2− anterior	15.7, −16.2, 1.7	1.4	40	130
6/VIM	A	12	1− 2c− 3a+	−14.5, −17.6, −0.64 −15.7, −16.4, 1.1 −15.6, −14.1, 2.0	1.5	80	100	11− ring	16.0, −13.9, 1.4	2.2	80	130
7/VIM	MS	18	3 ring (0.5)	−14.2, −15.2, 2.7	1.5	60	120	10− ring (0.4)	14.2, −15.8, 0.11	1.3	60	120

*Note*: Target: GPi = globus pallidus pars interna; VIM = ventral intermediate nucleus of thalamus. Lead type: MS = Medtronic SenSight lead with narrow contact spacing and Medtronic Percept; M = Medtronic 3389 lead with Medtronic PC; B = Boston Vercise Cartesia; A = Abbott 6172ANS with Infinity V2. LR = left–right; AP = anterior–posterior; SI = superior–inferior.

### 3.1 Neuropsychological Testing

Preoperatively, all three patients in the GPi group had letter fluency scores within broad average limits for age (11th to 18th percentile range bracket or above). Postoperatively, one declined from the 29th–40th percentile range to the 11th–18th percentile range, one stayed within the 11th–18th percentile range, and the third declined from the 82nd to 89th percentile range to the 60th to 71st percentile range (Table S1). Similarly, neither visuomotor scanning speed on Part A of the Trail Making test nor scanning speed with the added requirement of mental set alternation (Part B of the Trail Making test) showed clinically meaningful changes.

### 3.2 Primary Voice Outcomes

Overall descriptive statistics between GPi‐DBS and VIM‐DBS are reported in Table 3.

**TABLE 3:** Descriptive statistics of primary and secondary variables before (pre) and after (post) globus pallidus pars interna (GPi) (*n* = 4) and ventral intermediate nucleus of the thalamus (VIM) (*n* = 3) deep brain stimulation (DBS).

Variables	Group	Pre‐DBS		Post‐DBS	
		Mean	SD	Mean	SD
Percentage voicing (%)	GPi	80.81	20.12	99.20	1.34
	VIM	96.45	5.83	100	0
Cepstral peak prominence (dB)	GPi	9.79	4.27	12.45	1.98
	VIM	15.89	2.57	16.99	2.15
Tremor rate (Hz)	GPi	2.40	1.71	2.56	2.01
	VIM	3.89	0.33	2.78	0.83
Duration of voice breaks (ms)	GPi	0.09	0.08	0	0
	VIM	0.03	0.01	0	0
Number of voice breaks	GPi	1.30	1.25	0.11	0.33
	VIM	0.67	1.12	0	0
Extent of frequency modulation (%)	GPi	8.65	8.98	6.29	5.73
	VIM	7.07	6.63	3.61	3.06
Extent of intensity modulation (%)	GPi	9.01	6.86	2.48	2.20
	VIM	3.94	2.79	1.47	0.67
Voice handicap index total score	GPi	86.50	12.56	53.25	45.95
	VIM	—	—	—	—

Abbreviation: SD, standard deviation.

#### 3.2.1 Within‐Group Comparison

Estimated differences (Est), standard errors (SE) and *p*‐values are reported from linear mixed effects models comparing time (pre‐ to postoperative) by group (GPi and VIM) along with Cohen's dz effect size (Table 4). Within the GPi‐DBS group, acoustic measures of percentage voicing (Est = 18.89, SE = 4.01, *p* < 0.001, dz = 1.02), CPP (Est = 2.54, SE = 0.94, *p* = 0.03, dz = 1.01), duration of voice breaks (Est = −0.09, SE = 0.02, *p* < 0.001, dz = −1.37), number of voice breaks (Est = −1.21, SE = 0.32, *p =* 0.002, dz = −0.71), and the extent of intensity modulation (Est = −6.5, SE = 1.06, *p* < 0.001, dz = −1.44) showed trends for large improvements following treatment with GPi‐DBS. Although the effect size corresponds to a medium magnitude, the calculations are likely unreliable with high uncertainty due to small *n*. Further the changes in the extent of fundamental frequency modulation (Est = −2.46, SE = 1.67, *p* = 0.21, dz = −0.63), and tremor rate (Est = 0.21, SE = 0.23, *p* = 0.39, dz = 0.50) were not statistically significant. Within the VIM‐DBS group, tremor rate (Est = −1.11, SE = 0.23, *p* < 0.001, dz = −1.09) showed trends for significant improvement following treatment with VIM‐DBS. Improvements in the extent of frequency modulation (Est = −3.46, SE = 1.71, *p* = 0.09, dz = −0.79), extent of intensity modulation (Est = −2.47, SE = 1.08, *p* = 0.06, dz = −1.07), percentage voicing (Est = 3.55, SE = 4.09, *p* = 0.39, dz = 0.58), CPP (Est = 1.1, SE = 0.96, *p* = 0.30, dz = 0.45), duration of voice breaks (Est = −0.03, SE = 0.02, *p* = 0.19, dz = −0.58), and number of voice breaks (Est = −0.67, SE = 0.33, *p* = 0.08, dz = −0.58) showed smaller changes (medium effect size) in the VIM group that were not significant.

**TABLE 4:** Results from linear mixed models with comparisons within and between groups globus pallidus pars interna (GPi) (*n* = 4) and ventral intermediate nucleus of the thalamus (VIM) (*n* = 3) deep brain stimulation (DBS).

Variable	Group	Pre to post change within‐group							Differences between groups						
		Est	SE	LCL	UCL	*t*	df	FDR *p*	Est	SE	LCL	UCL	*t*	df	FDR *p*
Percentage voicing (%)	GPi	18.89	4.01	11.04	26.74	4.7	28.1	< 0.001	15.34	5.73	4.11	26.56	2.7	28.1	0.030
	VIM	3.55	4.09	−4.47	11.58	0.9	28.0	0.393							
CPP (dB)	GPi	2.54	0.94	0.70	4.39	2.7	28.1	0.029	1.44	1.35	−1.20	4.08	1.1	28.1	0.343
	VIM	1.10	0.96	−0.79	2.99	1.1	28.0	0.303							
Tremor rate (Hz)	GPi	0.21	0.23	−0.24	0.66	0.9	28.0	0.388	1.32	0.33	0.68	1.96	4.0	28.0	0.003
	VIM	−1.11	0.23	−1.57	−0.65	−4.8	28.0	< 0.001							
Duration voice breaks (ms)	GPi	−0.09	0.02	−0.13	−0.05	−4.5	28.2	< 0.001	−0.06	0.03	−0.11	−0.01	−2.0	28.1	0.058
	VIM	−0.03	0.02	−0.07	0.01	−1.5	28.1	0.191							
No. voice breaks	GPi	−1.21	0.32	−1.84	−0.57	−3.8	28.2	0.002	−0.54	0.46	−1.44	0.36	−1.2	28.1	0.343
	VIM	−0.67	0.33	−1.31	−0.02	−2.0	28.1	0.088							
Extent frequency modulation (%)	GPi	−2.46	1.67	−5.73	0.81	−1.5	28.0	0.207	1.00	2.39	−3.68	5.68	0.4	28.0	0.679
	VIM	−3.46	1.71	−6.80	−0.12	−2.0	28.0	0.088							
Extent intensity modulation (%)	GPi	−6.50	1.06	−8.57	−4.42	−6.1	28.0	< 0.001	−4.02	1.51	−6.99	−1.05	−2.7	28.0	0.030
	VIM	−2.47	1.08	−4.60	−0.35	−2.3	28.0	0.064							
VHI total	GPi	−33.25	18.43	−69.40	2.87	−1.8	3.0	0.211	—	—	—	—	—	—	—

*Note*: Estimated means (EST), standard error (SE), lower confidence level (LCL), upper confidence level (LCL), *t*‐test, degrees of freedom (df), and Benjamini–Hochberg FDR method used to determine significance after controlling for multiple comparisons across the 15 within‐group comparisons and seven between‐group comparisons.

Abbreviations: CPP, cepstral peak prominence; no, number; VHI, voice handicap index.

#### 3.2.2 Between‐Group Comparison

**FIGURE 2:**
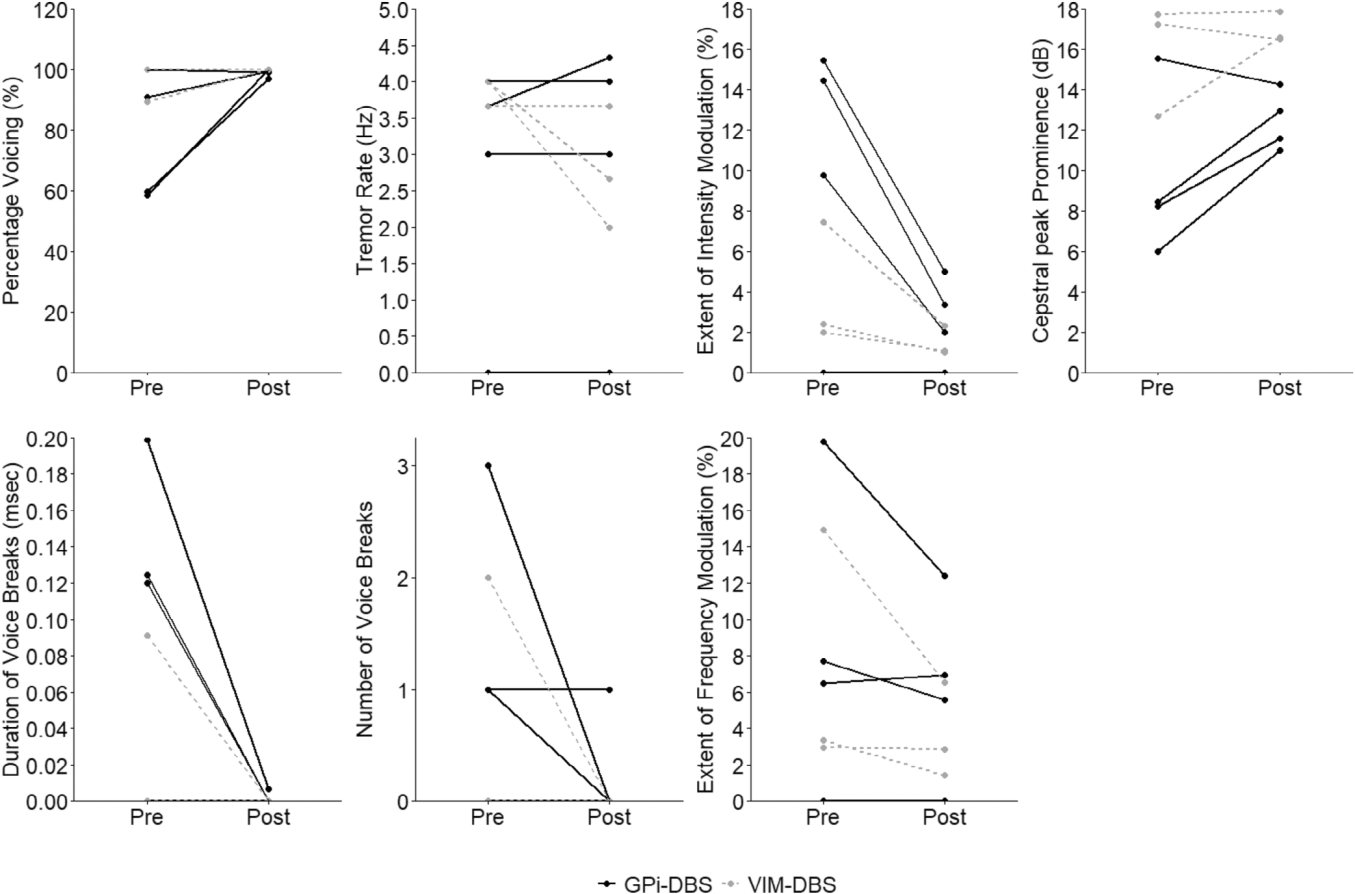
Spaghetti plots illustrating individual patient trajectories for each dependent variable, showing pre‐ and post‐operative changes following deep brain stimulation (DBS). Each line represents a single patient, with changes plotted separately for those undergoing globus pallidus interna (GPi) DBS (*n* = 4) and ventral intermediate nucleus of the thalamus (VIM) DBS (*n* = 3). These plots highlight within‐subject variability and potential trends between GPi‐DBS and VIM‐DBS conditions.

The interaction of time by group tested whether the pre‐ to postoperative changes in acoustic voice measures were significantly different between GPi‐DBS and VIM‐DBS groups. Percentage voicing (Est = 15.34, SE = 5.73, *p* = 0.03, *d* = 1.13) (Figure 2), extent of intensity modulation (Est = −4.02, SE = 1.51, *p* = 0.03, *d* = −1.20) showed numerically larger but not statistically significant changes in patients treated with GPi‐DBS compared to VIM‐DBS, whereas tremor rate (Est = 1.32, SE = 0.33, *p =* 0.003, *d* = 1.73) showed greater improvement after VIM‐DBS compared to GPi‐DBS. Duration of voice breaks showed trends for greater improvement with GPi‐DBS ((LS) *M* = −0.06, SE = 0.03, *p* = 0.06, *d* = −1.15) with large effect size, but it did not meet significance criteria after the FDR adjusted criteria. The following measures were not significantly different between groups: CPP (Est = 1.44, SE = 1.35, *p* = 0.34, *d* = 0.67), number of voice breaks (Est = −0.54, SE = 0.46, *p* = 0.34, *d* = −0.26).

### 3.3 Secondary Voice Outcome

**FIGURE 3:**
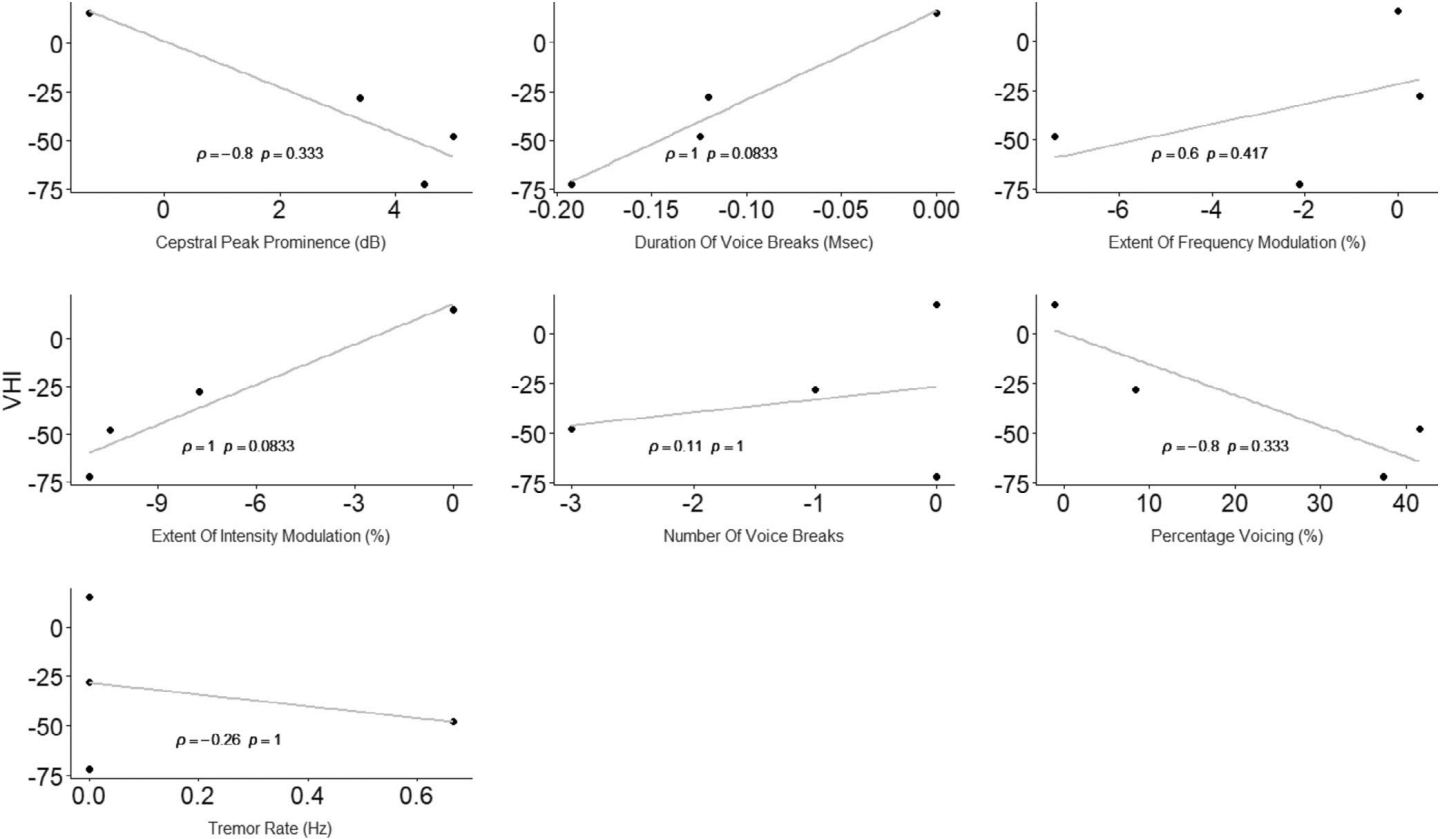
Correlation plots showing the relationship between changes in each dependent variable and changes in voice handicap index (VHI) total scores in patients undergoing globus pallidus interna (GPi) DBS (*n* = 4). Each point represents an individual patient, illustrating potential associations between clinical outcomes and perceived voice handicap.

Exploratory Spearman correlations were large but not significant between change in total voice handicap index score and the following variables: (a) the changes in the duration of voice breaks (rho = 1.00, *p* = 0.083, *n* = 3), (b) changes in the extent of intensity modulation (rho = 1.00, *p* = 0.083, *n* = 3), (c) changes in percentage voicing (rho = −0.80, *p* = 0.33, *n* = 4), and (d) CPP (rho = −0.80, *p* = 0.333, *n* = 4) in patients undergoing GPi‐DBS (Figure 3). The correlations were small for tremor rate (rho = −0.26, *p* = 1.00, *n* = 4), number of voice breaks (rho = 0.11, *p* = 1.00, *n* = 4), and extent of frequency modulation (rho = 0.60, *p* = 0.42, *n* = 4).

Adverse events observed during the study are summarized in Table 5.

**TABLE 5:** Reported adverse events of the patients undergoing deep brain stimulation (DBS) surgery of the globus pallidus interna (GPi) and ventral intermediate nucleus of the thalamus (VIM).

Patient #	Sex	Age at surgery	Handedness	Primary diagnosis	DBS surgery	Reported adverse events
1	F	73	R	ADLD/vocal tremor	GPi	Asymptomatic superficial hemorrhage on delayed MRI which was not present on the initial postoperative CT scan. No further treatment was required, as this adverse event is known to occur in DBS surgery (estimated 2% risk)
2	M	58	L	ADLD	GPi	None
3	F	76	R	ADLD/vocal tremor	GPi	Prolonged hospital stay for nausea and headaches, of 4 days versus the general range of 1–2 days
4	F	70	R	ADLD/vocal tremor	GPi	None
5	F	69	L	ADLD/ET/vocal tremor	VIM	None
6	F	70	L	ADLD/ET/vocal tremor	VIM	None
7	F	82	R	ADLD/ET/vocal tremor	VIM	None

Abbreviations: ADLD, adductor laryngeal dystonia; CT, computerized tomography; ET, essential tremor; F, female; M, male.

## 4 Discussion

To our knowledge, this is the largest series looking at effects of GPi‐DBS and VIM‐DBS on ADLD. Our study represents the largest series to date on *bilateral* DBS for ADLD. DBS is a mainstay in the surgical treatment of movement disorders [6]; the fact that patients who underwent either GPi‐ or VIM‐DBS improved after surgery supports the involvement of impaired basal ganglia‐subcortical motor networks in LD [6], complementing rather than excluding the role of abnormal sensory processing [46, 47, 48], in the disorder's complex pathophysiology. As with functional neurosurgical intervention for dystonia using the same target, GPi‐DBS for LD did not impair psychomotor speed or executive function [49].

### 4.1 Primary Outcome Variables

Through detailed objective acoustic analyses, we identify distinct acoustic measures that may reflect the voice changes after GPi‐DBS and VIM‐DBS. The GPi‐DBS treatment demonstrated preliminary differential effects in several voice parameters. The substantial change in vowel voicing (percentage voicing) and CPP suggests that GPi‐DBS has a preliminary significant impact on establishing continuous voicing. This finding is consistent with our previous finding in a single patient [19]. The trend in reduction of number and duration of voice breaks also indicates improved continuity of voicing. Together, these findings suggest that GPi‐DBS may improve overall motor control of speech production by improving speech fluency and reducing voice breaks that are often seen in patients with ADLD [1]. The preliminary trend of GPi‐DBS on intensity modulation demonstrates a reduction in the ability to modulate vocal intensity of tremor. This suggests that GPi‐DBS may improve voice by reducing tremor intensity and minimizing or eliminating voice breaks, without affecting tremor rate [19]. The results of the current study, in *n* = 7, align with our earlier observations from two patients, indicating that bilateral GPi‐DBS may lead to a greater reduction in the extent of fundamental frequency/intensity modulation compared to bilateral VIM‐DBS [18], however the tremor rate remains unchanged [19]. While tremor and voice breaks can both impact voice quality, it is important to recognize that they are distinct phenomena associated with different underlying disorders, vocal tremor and ADLD, respectively. Tremor typically presents as rhythmic movements affecting the voice, whereas voice breaks in ADLD are characterized by intermittent spasmodic interruptions during voiced speech due to abnormal muscle contractions. The observation that GPi‐DBS may reduce voice breaks could suggest that stimulation could modulate dystonic laryngeal activity directly, in addition to its impact on tremor severity. The observed GPi‐DBS and VIM‐DBS differences may partly reflect confounding by indication, as patients with more dystonia‐dominant versus tremor‐dominant phenotypes are typically directed toward different stimulation targets. Accordingly, these findings should be interpreted as hypothesis‐generating and consistent with existing phenotype‐based target selection, rather than as definitive evidence of target‐specific efficacy. Further research is needed to clarify whether this effect reflects a broader therapeutic influence of GPi‐DBS on both tremor and dystonia‐related symptoms, or whether the improvement in voice breaks is secondary to the reduced tremor intensity.

In contrast to the changes observed in voice with GPi‐DBS, the rate of vocal tremor was significantly reduced in patients who underwent VIM‐DBS. This is consistent with our prior findings on one patient with ADLD [19] and 10 patients with medically refractory essential tremor who were treated with VIM‐DBS [18]. VIM‐DBS also resulted in a reduced extent of intensity modulation. Although the reduction in frequency modulation was not statistically significant, a trend toward reduction with a moderate effect was observed, warranting further investigation. This may reflect a weaker or a more variable effect in this parameter. These findings align with previous reports of significant reduction in the modulation of frequency and intensity in patients with essential vocal tremor treated with VIM‐DBS [50]. However, unlike the Erickson‐DiRenzo [50] study, which focused solely on vocal tremor, all patients in our study who underwent VIM‐DBS were also diagnosed with ADLD in addition to vocal tremor. Similar effects have been reported in Canada and other institutes in the US, where improvements in voice were observed on the auditory‐perceptual evaluation in several case series with patients who underwent bilateral VIM‐DBS [51, 52].

### 4.2 Secondary Outcome Variable

The finding of a preliminary strong relationship between improvements in the duration of voice breaks and intensity modulation, along with a consistent change in the total voice handicap index score, indicates an overall improvement in the perceived impact of voice‐related quality of life in patients with GPi‐DBS. This association suggests that changes in the duration of voice breaks and extent of intensity modulation may be related to perceived improvement captured by the total voice handicap index score; however, no direct causal relationship can be inferred from the current data. The specific effects on voice breaks and intensity modulation may point to how GPi‐DBS influences the motor control of voice, resulting in a more stable and less strained voice. This highlights GPi‐DBS as potentially more effective than VIM‐DBS for addressing voice breaks and extent of intensity modulation aspects of voice dysfunction in patients with ADLD. The factors contributing to patient satisfaction and quality of life improvements after VIM‐DBS are unclear and should be explored in future studies.

### 4.3 Limitations

Although this study represents the largest series on bilateral DBS surgery to treat ADLD, the sample size remains small; more research is needed, ideally via a multicenter, randomized, controlled trial, to establish generalizability. The analyses are subject to uncertainty and should be interpreted with caution. Additionally, patients who receive prolonged VIM‐DBS therapy [53] may develop habituation or tolerance to stimulation after years of treatment. While this is well described in essential tremor of the hands, it has not been studied in vocal tremor, and it is not known whether the same phenomenon would occur in patients with ADLD treated with VIM‐DBS. Further longitudinal studies are needed. Future studies should include measurement of voice breaks during sentence production to further validate and extend these findings, as this is the established approach to assess LD symptoms as per Ludlow et al. [54]. While this study compares GPi‐ and VIM‐DBS for ADLD, it was not a randomized trial. Patients treated with VIM‐DBS also had disabling hand tremor, so it is possible that the underlying pathophysiology of their ADLD is different than that of the GPi‐DBS treated ADLD patients.

## 5 Conclusion

Our study represents the first preliminary series showing target‐specific patterns of GPi‐DBS and VIM‐DBS surgery in treating symptoms of ADLD. Our findings highlight GPi‐DBS as potentially more effective for addressing voice breaks and particularly beneficial for patients with ADLD without tremor. In contrast, VIM‐DBS resulted in significant and strong improvements in tremor rate and intensity modulation, suggesting the potential of VIM‐DBS as a treatment for patients with ADLD with tremor. While randomized controlled trials are necessary to further investigate these effects, our findings point to DBS as a potentially novel treatment modality for patients suffering from two common variants of this often debilitating condition.

## Funding

This work was supported by the National Institutes of Health (UL1TR002529).

## Conflicts of Interest

Dr. S. Elizabeth Zauber has been a site investigator for registry trials with Abbott and Boston Scientific, and Medtronic. Dr. Kunal Gupta has been a site investigator for clinical trials and registries with Abbott and Boston Scientific, and has consulted for Aspen Neuroscience, Solid Biosciences, and NeuroOne.

## Supporting information


**Table S1:** Preoperative and postoperative results of the neuropsychological tests performed. COWA = Controlled Oral Word Association Test; Trails A = Trail Making Test Part A; Trails B = Trail Making Test Part B, Raw = raw scores for COWA (words produced in 60 s), and the Trail Making Tests (seconds to complete); SS = scaled scores (mean = 10, SD = 3) from the Minnesota Older Adults Normative Study, with conversion to percentile ranges.

## Data Availability

The data that support the findings of this study are available from the corresponding author upon reasonable request.
