# Supplementary material for: Effects of Deep Brain Stimulation on Adductor Laryngeal Dystonia
Source: Laryngoscope. 2026 Feb 16;136(7):3085–96. doi: 10.1002/lary.70441 (PMC13253171; doi:10.1002/lary.70441)
Supplement: Supplementary file 1 — Table S1: Preoperative and postoperative results of the neuropsychological tests performed. COWA = Controlled Oral Word Association Test; Trails A = Trail Making Test Part A; Trails B = Trail Making Test Part B, Raw = raw scores for COWA (words produced in 60 s), and the Trail Making Tests (seconds to complete); SS = scaled scores (mean = 10, SD = 3) from the Minnesota Older Adults Normative Study, with conversion to percentile ranges. [file LARY-136-3085-s001.docx]

| **Pre-Operative** | | | | | | | | | |
| --- | --- | --- | --- | --- | --- | --- | --- | --- | --- |
| **Subject** | **COWA** | | | **Trails A** | | | **Trails B** | | |
|  | **Raw** | **SS** | **Percentile Range** | **Raw** | **SS** | **Percentile Range** | **Raw** | **SS** | **Percentile Range** |
| 1 | 24 | 7 | 11^th^-18^th^ | 55 | 7 | 11^th^-18^th^ | 111 | 9 | 29^th^-40^th^ |
| 2 | 48 | 13 | 82^nd^-89^th^ | 16 | 17 | 99^th^ | 60 | 11 | 60^th^-71^st^ |
| 3 | 30 | 9 | 29^th^-40^th^ | 37 | 10 | 41^st^-59^th^ | 96 | 10 | 41^st^-59^th^ |
| **Post-Operative** | | | | | | | | | |
| **Subject** | **COWA** | | | **Trails A** | | | **Trails B** | | |
|  | **Raw** | **SS** | **Percentile Range** | **Raw** | **SS** | **Percentile Range** | **Raw** | **SS** | **Percentile Range** |
| 1 | 23 | 7 | 11^th^-18^th^ | 43 | 9 | 29^th^-40^th^ | 148 | 8 | 19^th^-28^th^ |
| 2 | 40 | 11 | 60^th^-71^st^ | 18 | 15 | 95^th^-97^th^ | 68 | 10 | 41st-59^st^ |
| 3 | 24 | 7 | 11^th^-18^th^ | 27 | 13 | 82^nd^-89^th^ | 111 | 10 | 41^st^-59^th^ |

**Supplemental Material**: Pre-Operative and Post-Operative results of the neuropsychological tests performed. ***Notes***: COWA= Controlled Oral Word Association Test; Trails A= Trail Making Test Part A; Trails B= Trail Making Test Part B, Raw = raw scores for COWA (words produced in 60 sec), and the Trail Making Tests (seconds to complete); SS= scaled scores (mean= 10, SD=3) from the Minnesota Older Adults Normative Study, with conversion to percentile ranges.
